# Atypical language lateralization in positive schizotypy and modulating effects of the menstrual cycle

**DOI:** 10.1016/j.cpnec.2025.100291

**Published:** 2025-04-02

**Authors:** Helene Hjelmervik, Josef J. Bless, Julien Laloyaux, Kenneth Hugdahl, Markus Hausmann

**Affiliations:** a School of Health Sciences, Kristiania University College, Bergen, Norway; b Institute of Medical Psychology, LMU Munich, Munich, Germany; c Psyliège Psychological Consultation Center, Liège, Belgium; d Department of Biological and Medical Psychology, University of Bergen, Norway; e Division of Psychiatry, Haukeland University Hospital, Bergen, Norway; f Department of Radiology, Haukeland University Hospital, Bergen, Norway; g Department of Psychology, Durham University, United Kingdom

**Keywords:** Schizotypy, Language lateralization, Cognitive control, Dichotic listening, Menstrual cycle phase

## Abstract

Previous studies have shown atypical language asymmetry in male participants with high schizotypy traits, but in female participants the pattern is less clear. Such sex differences could suggest a role of sex hormones, especially since hemispheric asymmetries have been shown to change across the menstrual cycle. By modulating attention in a consonant-vowel dichotic listening test, the current study aimed to investigate language lateralization (ear advantage of non-forced condition) in high vs low positive-schizotypy and the role of the menstrual cycle. In addition, we aimed to replicate menstrual cycle effects on the left attention condition. Thirty-nine female participants were tested in the menstrual (low estradiol) or follicular (high estradiol) cycle phase. Women tested in the follicular phase were found to perform better on the left attention condition, indicating enhanced cognitive control. In the non-forced condition, the high positive-schizotypy group showed increased right hemispheric involvement during the follicular phase relative to the menstrual phase; whereas an increase in left hemispheric dominance was seen in the low positive-schizotypy group during this cycle phase. The results suggest an underlying difference in lateralization between low and high positive-schizotypy that is enhanced by gonadal hormones, perhaps through altered interhemispheric inhibition. Overall, the study suggests that the atypical language lateralization in high schizotypy individuals is highly flexible and dependent on the hormonal milieu, and could potentially be related to neuroprotective effects of estradiol.

## Highlights

• High schizotypy traits were found related to atypical language lateralization.
• Atypical lateralization in high schizotypy was modulated by menstrual cycle phase.
• All participants showed enhanced cognitive control in the follicular cycle phase.
• The enhanced cognitive control was related to estradiol.

## 1 Introduction

The dichotic listening task [[1], [2], [3]] is a well-established paradigm to investigate language lateralization. In the Bergen version of the dichotic listening paradigm [4,5] meaningless consonant-vowel syllables (CV; e.g.,/ba/,/pa/) are presented simultaneously to the right and left ear. In the non-forced (NF) condition of the task, the participants are instructed to report the syllable that is heard best or clearest. Participants more often report the stimulus presented to the right ear; a phenomenon called the right ear advantage (REA). It has been interpreted to indicate the dominance of the left-hemisphere in speech perception [6,7], and is based on the preponderance of the contralateral pathways (i.e. neuroanatomical model; Kimura [6]). The dichotic listening task has been validated against the Wada procedure, which uses unilateral intracarotid amobarbital (often in combination with EEG) to determine the dominant hemisphere for speech, by asking the patient to count from one and up while the right and subsequently the left hemisphere is being sedated [8].

However, the REA can be modulated by asking participants to explicitly shift their attention to the left or right ear, thus adding a top-down cognitive effect to the bottom-up stimulus effect [5,9]. In the forced-right (FR) condition, participants are asked to report syllables from the right ear and typically show an enhanced right-ear advantage; while in the forced-left (FL) condition participants are asked to report from the left ear and usually show a reduced REA or even a left-ear advantage (LEA [10]). The FR condition is thought to require *non-executive attention* and the FL condition, additionally, *executive control* processes [[10], [11], [12]]. In the FR condition, the participant is being supported by the bottom-up effect as they report syllables from the dominant/preferred ear. In contrast, in the FL condition the participant is asked to report the non-dominant stimulus from the left ear and inhibit the dominant right ear stimulus. This requires executive or cognitive control resources to overcome the otherwise dominating stimulus driven bottom–up effect [10]. Although the individual responses for the FR and FL conditions clearly depend on individual degree of language lateralization, these two conditions involve different aspects of top-down resources as compared to the NF condition which reflects unbiased language lateralization. Hence, the three instruction conditions of the Bergen dichotic listening paradigm reflect different cognitive processes [9].

Reduced or reversed lateralization of language perception has been associated with clinical groups, such as schizophrenia patients (for a review see Ref. [13]) Some studies confirms this pattern at a sub-clinical level also in high schizotypy non-patients [14,15], although to a lesser extent and with less consistency across studies (Castro and Pearson). Schizotypy refers to trait characteristics in the normal population that resembles to some extent symptoms of schizophrenia. The Schizotypy Personality Inventory (SPQ) is a well-established scale that was developed on the basis of the DSM-III criteria for schizotypal personality disorder. The SPQ exists in different versions in which a three-factor structure of the inventory has repeatedly been validated [[16], [17], [18]]. The three factors refer to three different schizotypy traits: (1) The cognitive-perceptual trait corresponding to positive symptoms (e.g. hallucinations) in schizophrenia, (2) the interpersonal dimension reflecting negative symptoms (e.g. social withdrawal), and (3) the disorganized dimension reflecting cognitive symptoms. Traits can be assessed separately or as a composite score, with high scores reflecting a high presence of schizotypy traits (i.e., high schizotypy) and low scoring reflecting low degree of schizotypy (e.g. Ref. [19]). Especially positive symptoms have been related to reduced language lateralization in schizophrenia (e.g. Ref. [20]). This has also been shown in a dichotic listening study, where only hallucinating patients showed reduced REAs [21]. It has been proposed that an abnormality in the left hemisphere can account for both the atypical lateralization and the auditory hallucinations [12]. Therefore, the current study focused on positive symptoms when studying language lateralization in schizotypy, as suggested by Castro and Pearson [22].

Language lateralization has been studied with dichotic listening tasks in high schizotypy male and female participants. Abnormal language lateralization, with more right shifted language, was shown specifically in a NF CV-syllables dichotic listening task in high schizotypy males [23]. High and low schizotypy was here determined by a composite score across three schizotypy scales (respectively >1 SD above mean and <½ SD below mean). Similarly, Voglmaier (2009) found reduced REA for a male group with schizotypal personality disorder (DSM-IV) as compared to the control group in a competing sentence forced attention task, whereas the female group did not differ from the controls. In a mixed gender sample Castro and Pearson [22] did not find altered REA in a NF word dichotic listening task in high vs low schizotypy individuals as categorized by the median of the total SPQ score. The difference between male and female high-schizotypy samples [24] could suggest that sex hormones play a role.

Hemispheric asymmetries have been found to change across the menstrual cycle with alternating levels of estrogen and progesterone (e.g. [25,26]). The original model, which is mainly based on visual half field paradigms, suggests reduced hemispheric asymmetry in response to circulating progesterone and estradiol (for a review see Hausmann and Bayer, 2010). However, in dichotic listening studies, findings are inconsistent. Three different menstrual cycle phases, characterised by different hormone levels, have been studied in this respect: The menstrual phase (day 1–5) is characterised by low estrogen and progesterone levels. The (late) follicular cycle phase (day 7–12) is characterised by high estrogen and low progesterone levels. The midluteal phase (day 20–22) is characterised by high estrogen and progesterone levels. Some studies have found stronger left hemispheric language lateralization (NF-condition) in the follicular [27] and luteal phase [[27], [28], [29]] as compared to the menstrual phase. Other studies found the FL-condition to be affected in the follicular phase, but not the NF-condition [30,31], suggesting that sex hormones do not affect language lateralization but rather improve cognitive control abilities. Furthermore, the increased number of FL left-ear reports in Hjelmervik et al.‘s (2012) study was found related to the follicular increase in estradiol.

While most of these studies included only right-handed (except Morris et al.) natural cycling women, there are also differences in methodology that might have contributed to some of the inconsistencies across studies. The studies vary in the cycle phases tested and only two studies have used hormone assays to validate cycle phases ([27] (blood); [30] (saliva)). In addition, studies differed in the verbal stimuli used and the analyses. While Cowell et al. Hjelmervik et al., and Morris et al. used CV stimuli, Sanders and Wenmonth included additional emotional and neutral word-stimuli. Wadnekar et al. used CV-stimuli but merged responses from the three conditions (NF, FR and FL) in the analysis, and therefore the DL bias was most likely confounded by the attention condition across the menstrual cycle.

Another study [32], which again deviates in methodological approach by splitting participants into high and low estradiol groups (irrespective of cycle phase), found that high estradiol was related to lower degree of asymmetry in the NF condition. The rightward shift in laterality with high estradiol has later been argued to reflect a mechanism of neuroprotection in which additional resources of the right hemisphere is recruited [33]. Protective effects of estradiol in schizophrenia are well documented and was recently also shown to affect hallucination tendencies in healthy participants (Hjelmervik et al., 2023), however, the underlying mechanisms for neuroprotective effects are still poorly understood and shifts in hemispheric asymmetries could play a role.

The current study therefore investigated the REA in dichotic listening in women with high vs low schizotypy traits during the high estradiol follicular phase vs the low estradiol menstrual phase. In addition, we aimed to replicate the most frequent ([30,31]: versus [27]) previous findings of menstrual cycle effects on dichotic listening for the given cycle phases. Therefore, data were analyzed in two steps; without and with taking schizotypy groups into account. We hypothesized (H1) that women tested in the follicular cycle phase would perform better on the FL-condition as reflected by more left-ear reports/fewer right ear-reports; but no difference between the cycle phases would be evident in the FR- and NF-conditions [30,31]. It was also hypothesized (H2) that estradiol would be positively related to more leftward ear-score in the FL-condition [30]. Further, reduced left hemispheric dominance for language processing (NF-condition) in the high schizotypy group as compared to the low schizotypy group was hypothesized (H3; [23], and that this atypical language lateralization would be more prominent (less lateralized) in the high estradiol cycle phase (H4; [32,33].

## 2 Methods

### 2.1 Participants

Thirty-nine right-handed healthy women (out of fifty-one originally tested: see section on hormone assays for exclusion criteria) with mean age of 22.1 years of age (SD = 4.47, range 18–34) were tested on the Bergen forced-attention dichotic listening task either during the menstrual cycle phase (i.e. early follicular phase; cycle day 2–4) or during the follicular cycle phase (i.e. late follicular phase; cycle day 9–12) – representing a between-subject design. A power analysis based on previous study by Hjelmervik et al. [30] on menstrual cycle effects in dichotic listening, FL (effect size: ƞ^2^ = 0.12), suggested a total sample of 36 women (G∗power with a power of 0.90, alpha 0.05, ANOVA within-between interaction effect, option: effect size specification according to G-power 3.0., corr among repeated measures 0.5, nonsphericity correction 1).

Only participants who had regular menstrual cycles with mean cycle length between 26 and 32 days, and the right hand as preferred writing hand were included in the study. Further, they had to answer negative to the following: having been pregnant for the last six months; using hormonal contraceptives or other hormone regulating medication during the last six months; having any psychiatric or neurological disease All participants had normal hearing, as established by a hearing test at the frequencies 500, 1000, 2000, 4000 and 8000 Hz. Participants were tested in either the menstrual phase (day 2–4) or the follicular phase (day 9–12). The backward-count method was used to estimate cycle phase. Data collection took place at the University of Bergen (Norway) and Durham University (UK), and ethical approval was obtained from the Regional Committee for Medical Research Ethics in Norway (2018/411), and the local ethics committee at the University of Durham, respectively. Participants gave their informed consent according to the Declaration of Helsinki.

### 2.2 Hormone assays

Assessment of ovarian hormones from saliva is a convenient and well-established methodology that allows for measuring the free circulating (active) hormones, which is well correlated with the free fraction in serum [34]. Three saliva samples were collected from each participant, two before and one after the dichotic listening task. The participants were asked to refrain from drinks and food the last hour before the testing, and to rinse their mouth with water at arrival. For the collection of saliva samples, SaliCap (IBL International) were used. Luminescence ELISA assays were applied in the analysis of the saliva samples. An equal amount from each of the three samples were blended before analysis and analyzed for estradiol and progesterone. Hormone levels therefore represent an average of the three samples. The analyses of samples collected in Bergen and Durham took place in the laboratory at the Department of Biological and Medical Psychology at University of Bergen. Sensitivities for the analyzed steroids/assays are: Progesterone: Limit of Detection (LoD): 8,9 pg/ml,. 17-beta-Estradiol: LoD: 0,3 pg/ml (IBL International). Inter-assay coefficient of variation for progesterone was 18.4–23.4 (two levels), and for estradiol 11.7–20.8 (two levels). Before scheduling testing of participants, individual cycles were tracked for 2–3 months to estimate mean cycle length. When scheduling testing of participants, the backward counting method (e.g. Ref. [35]) was used to maximize the likelihood of testing the women in the cycle phases of interest. The date for day-1 in the last menstruation was used to calculate the next menstrual onset according to individual cycle length, and from there we counted backwards 17–20 days to estimate the (late) follicular cycle phase. Hormone levels were used to verify menstrual cycle phases. Exclusion criterion was based on the general principles of progesterone being low in the menstrual and follicular cycle phase. To exclude participants erroneously tested in the luteal phase, IBL's instruction sheet for expected ranges pr cycle phase were used, suggesting that a progesterone level of 127 pg/ml or above indicates luteal cycle phase. For the current sample only participants with progesterone levels below 127 pg/ml were included. In addition, two outliers of estradiol as identified with SPSS, were excluded (which also falls outside the expected range of estradiol in the menstrual phase according to IBL instruction sheet for estradiol; See Supplementary material). In total, eight participants were excluded based on hormone levels. It should be noted that in a related study we used stricter exclusion criteria [36]. As the current dichotic listening sample is a smaller sub-sample, a milder exclusion criterion was chosen to preserve power. In addition, one participant was excluded due to missing data, and another three due to irregular dichotic listening results – strong REA during FL and strong LEA during FR – suggesting low task compliance or that left and right headphones were erroneously swapped during testing. After exclusion, estradiol levels were shown to be significantly higher in the follicular cycle phase (M = 3.93 pg/ml, SD = 1.86) as compared to the menstrual phase (M = 3.03 pg/ml, SD = 1.18) as tested with a one-sided independent samples *t*-test (t (37) = 1.84, p = 0.04). For progesterone levels, there was no difference between the follicular (M = 42.87 pg/ml, SD = 23.02) and menstrual (M = 47.64 pg/ml, SD = 28.10) cycle phases (t (37) = 0.58, p = 0.28). The high (M = 3.95 pg/ml, SD = 1.66) and low (M = 3.11 pg/ml, SD = 1.55) positive-schizotypy groups did not significantly differ in estradiol levels on a two-sided independent *t*-test (t (37) = -1.63, p = 0.11). Neither was there a significant difference in progesterone levels between high (M = 47.55 pg/ml, SD = 26.58) and low (M = 42.21 pg/ml, SD = 23.89) positive-schizotypy group (t (37) = -0.66, p = 0.51).

### 2.3 Materials

#### 2.3.1 Dichotic listening

The Bergen Dichotic Listening test [11] was administered through the iDichotic app [37] on an iPod-touch in a laboratory setting. The stimulus set consisted of six consonant-vowel syllables,/ba/,/da/,/ga/,/ka/,/pa/,/ta/, recorded in a Norwegian male voice, and presented pairwise on each trial. Two syllables were presented simultaneously on each trial, one to the right and one to the left ear (dichotically) through headphones. All 36 pairwise combinations of the six syllables were used, including the six homonymic pairs (e.g.,/da/-/da/), which were excluded from the statistical analyses. The syllables had a duration of 400–500 ms and were presented with an inter-stimulus interval of 4000 ms. The participant responded to each pairwise presentation by touching the keyboard on the iPod screen displaying six buttons, one for each of the six syllables, which syllable she had perceived. The 36 stimulus pairs were presented three times in the three different attention instruction conditions (NF, FR, FL). All participants started with the NF condition where they were instructed to report the sound they “heard most clearly”. The NF condition was followed by the attentional conditions, FR and FL, and the order of these was counterbalanced across participants, with half receiving the FR condition first, the other half receiving the FL condition first. In the FR and FL instruction conditions, the participants were instructed to pay attention and only the syllable heard in the right or left ear, respectively. The number of correct left and right ear reports were scored separately for each condition (excluding the six homonymic pairs), and used for the calculation of laterality index (LI) (as a quantification of ear advantage) according to the formula: LI = [(RE − LE)/(RE + LE)] × 100. The laterality index reflects the percentage difference between correct left-ear and right-ear reports with positive values indicating a right-ear advantage/left hemisphere dominance, and negative values indicating a left-ear advantage/right hemisphere dominance. All included subjects had at least 60 % correct answers from either ear per condition, which is well above chance level (33,3 %).

#### 2.3.2 Schizotypy

The schizotypal personality questionnaire - brief revised updated (SPQ-BRU; Davidson et al., 2016) – was used to assess schizotypy. This version has been developed from the previous versions of the SPQ [38] and SPQ-B [18]. The SPQ-BRU consists of 32-items that are rated on a five-point scale ranging from ‘strongly disagree’ to ‘strongly agree’. Higher scores indicate stronger schizotypy. The SPQ-BRU has shown good reliability, divergent and discriminant validity [16]. The three-factor structure has been confirmed in the SPQ-BRU suggesting good construct validity. The three factors are as following: Cognitive/Perceptual (ideas of reference, suspiciousness, magical thinking and unusual perceptions), Interpersonal (no close friends; constricted affect, and social anxiety), and Disorganized (eccentric behaviour and odd speech). The current study used the scores on the Cognitive/Perceptual factor only, by summarizing the items for this factor. The cognitive/perceptual factor assessed with the SPQ-brief has shown high internal reliability (0.72), test-retest reliability (>0.86), and criterion validity (0.73) when compared to clinical interviews [18]. In the current sample, Cronbach's alpha (internal reliability) for the cognitive/perceptual factor was estimated to 0.76. The factor is referred to as positive-schizotypy in the following.

### 2.4 Statistical analyses

A 2(Cycle Phase) x3(Task) ANOVA was conducted in order to replicate previous studies (H1). In addition, a multiple regression analysis was done with the FL condition as dependent variable, and hormone levels – estradiol, progesterone and the interaction of the two – as regressors (H2). Finally, the High/Low Positive-Schizotypy variable was included, resulting in a 2(Cycle Phase) x 2 (High/Low Positive-Schizotypy) ANOVA for the NF condition to test H3 and H4. The high and low positive-schizotypy groups were determined by a median split (median = 31), resulting in four groups: (1) High positive-schizotypy, follicular phase (N = 10, age in years M = 21.7, SD = 5.41), (2) Low positive-schizotypy, follicular phase (N = 12, M = 24.00, SD = 5.78), (3) High positive-schizotypy, menstrual phase (N = 10, M = 20.40, SD = 1.82), (4) Low positive-schizotypy, menstrual phase (N = 7, M = 21.86, SD = 3.39). Age did not differ significantly between the groups (F (35,1) = 0.09, p = 0.77). Neither did the cycle phase groups significantly differ in positive-schizotypy scores within the low (t (17 = 0.26, p = 0.80; Menstrual: M = 25.71, SD = 3.63; Follicular: M = 26.25, SD = 4.71) and high (t (18) = 0.54, p = 0.59; Menstrual: M = 39.00, SE = 5.41; Follicular: M = 40.30, SD = 5.27) positive-schizotypy groups. In addition, exploratory analyses were done for the FR and FL condition with the same 2(Cycle Phase) x 2 (High/Low Positive-schizotypy) ANOVA set-up. And the analysis for the NF condition was re-run with the total schizotypy scores (including all three schizotypy factors). All post-hoc analyses were Bonferroni corrected.

## 3 Results

### 3.1 Cycle phase and dichotic listening

**Fig. 1:**
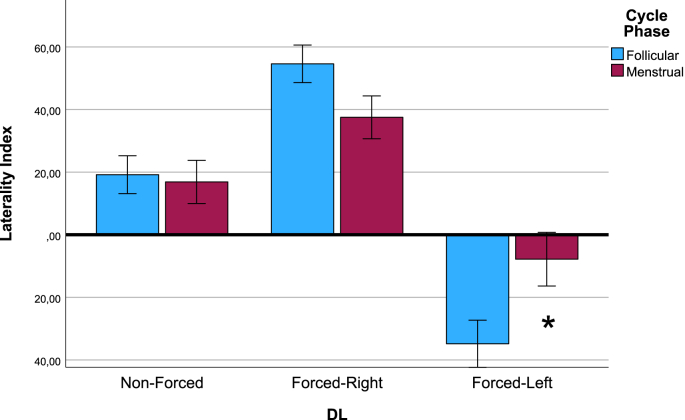
Laterality indexes for the three dichotic listening (DL) conditions for the follicular and menstrual cycle phase. Positive numbers on the laterality index refers to a right-ear advantage/left hemisphere dominance. Error bars refer to ± 1 standard error. ∗ refers to significant effect at 0.05 alpha level.

The 2(Cycle Phase) x3(Task) ANOVA resulted in a significant interaction effect between Cycle Phase and Task (F (2,36) = 4.28, p = 0.02, ƞ^2^ = 0.19; see Fig. 1). Post-hoc analysis to explore the interaction showed a significant difference between the menstrual (M = −7.81, SD = 38.74) and follicular (M = −34.82, SD = 32.41) cycle phase for the FL condition (t (37) = -2.37, p = 0.02, d = −0.77). This reflects a higher tendency to report from the left ear during the follicular cycle phase, indicating better cognitive control. No significant difference was found for the NF (t (37 = 0.25, p = 0.83, d = 0.08) condition between the menstrual (M = −16.88, SD = 19.18) and the follicular cycle phase (M = 19.19, SD = 33.75). No significant difference was found between the menstrual (M = 37.52, SD = 26.65) and follicular (54.60, SD = 29.32) cycle phase for the FR condition (t (37) = 1.87, p = 0.07, d = 0.60). In addition a main effect of task was found (F (2,36) = 39.37, p < 0.001, ƞ^2^ = 0.69), reflecting significant response differences between all three conditions/task instructions: a strong REA for the FR condition (M = 47.15, SD = 29.12), a moderate REA for the NF condition (M = 18.18, SD = 28.03), and a left ear advantage for the FL condition (−23.05, SD = 37.37).

The multiple regression analysis with hormone levels as regressors against the FL laterality index showed a significant effect of estradiol (F (1,35) = 5.64, p = 0.02, ƞ^2^ = 0.14), which mean, the higher the hormonal level the lower the laterality index (better performance). No other effects were significant (ALL F (1,35)<3.11, p > 0.09, ƞ^2^<0.08).

### 3.2 Positive-schizotypy, cycle phase and dichotic listening

#### 3.2.1 NF

**Fig. 2:**
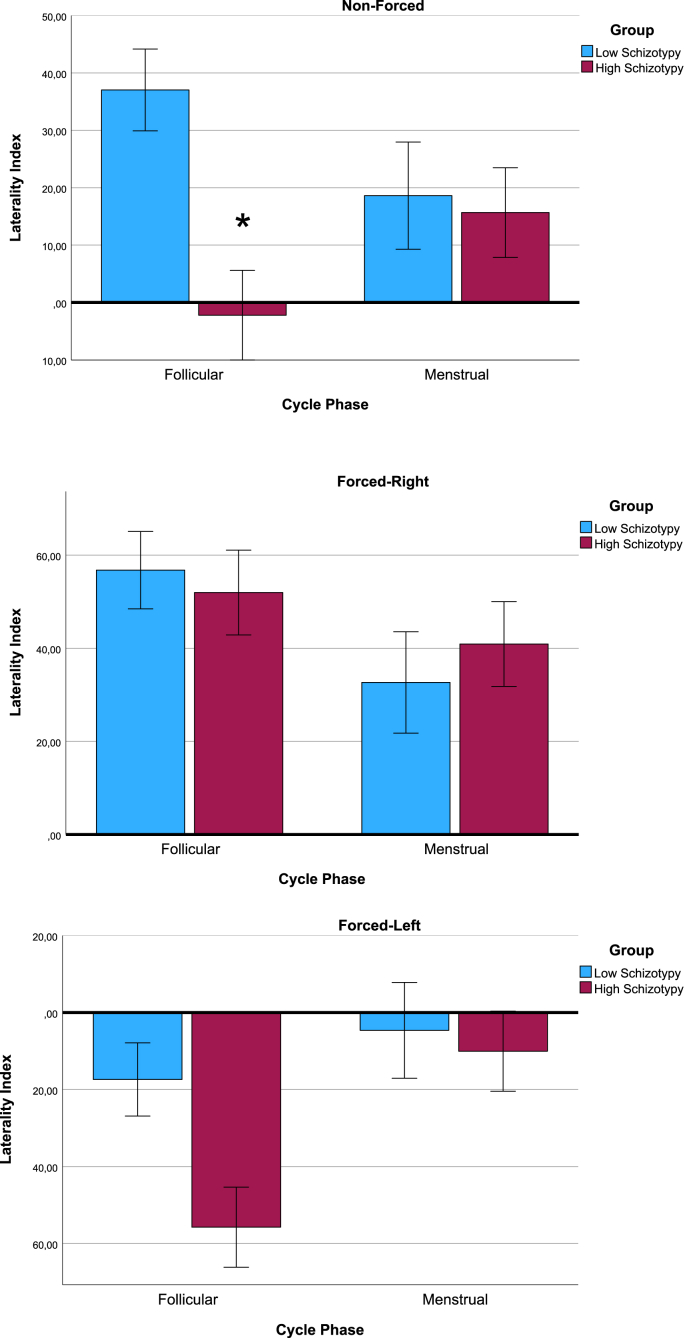
Laterality indexes for three dichotic listening conditions for the follicular and menstrual cycle phase in high- and low schizotypy groups. Positive numbers on the laterality index refers to a right-ear advantage/left hemisphere dominance. Error bars refer to ± 1 standard error. ∗ refers to significant effect at 0.05 alpha level.

The 2 (high/low positive-schizotypy) x 2(Cycle Phase) ANOVA for the NF condition showed a main effect of High/Low positive-schizotypy group (F (1,35) = 6.85, p = 0.01, ƞ^2^ = 0.16) in which the low positive-schizotypy group showed stronger REA, indicating a more pronounced leftward language lateralization (M = 30.25, SD = 24.56) than the high positive-schizotypy group (M = 6.72, SD = 26.77). In addition the ANOVA resulted in a significant interaction of Positive-schizotypy group and Cycle Phase (F (1,35) = 5.07, p = 0.03, ƞ^2^ = 0.13; see Fig. 2). Bonferroni post-hoc comparisons showed that only in the follicular cycle phase, there was a significant difference (t (20) = 3.29, p < 0.001, d = 1.41) between the high (M = −2.22, SD = 32.23) and low (M = 37.04, SD = 23.68) positive-schizotypy groups. In the menstrual phase, there was no significant difference (t (15) = 0.3, p = 0.81, d = 0.15) between the high (M = 15.66, SD = 7.81) and the low (M = 18.62, SD = 23.05) positive-schizotypy groups.

When the same analysis for the NF condition included the total schizotypy scores, no significant main effects or interaction were found, all F (1,35)<0.46, p > 0.5, d < 0.01.

#### 3.2.2 FL and FR

The exploratory analysis for the FL condition resulted in a marginal significant main effect of High/Low positive-schizotypy in which the High-positive-schizotypy group (M = −32.89, SD = 41.47) showed more negative laterality index as compared to the Low-positive-schizotypy group (M = −12.68, SD = 30.20). The effect of Cycle Phase seen in the first analysis persisted (F (1,35) = 7.40, p = 0.01, ƞ^2^ = 0.18). The analysis did not show a significant Cycle Phase x Positive-schizotypy interaction (F (1,35 = 2.35, p = 0.13, ƞ^2^ = 0.06).

The analysis for the FR condition showed no significant effects (ALL F (1,35)<3.75, p > 0.07, ƞ^2^<0.44).

## 4 Discussion

The study aimed to investigate whether menstrual cycle phase modulated atypical language lateralization in women with higher positive-schizotypy traits. In addition, the study aimed to replicate previous findings on (a) language lateralization and cognitive control across the menstrual cycle, and (b) atypical language lateralization in participants high in positive-schizotypy traits. The results showed higher performance on the FL cognitive control condition in the follicular cycle phase as compared to the menstrual cycle phase (see Fig. 1), and that higher estradiol levels contribute to the effect. There was no difference in the NF condition between the cycle phases (see Fig. 1). However, when the sample was divided into high- and low-positive-schizotypy groups, an interaction-effect of positive-schizotypy group and menstrual cycle phase appeared for the NF condition (see Fig. 2). Post-hoc testing suggested that the atypical (reduced) asymmetry for the high positive-schizotypy group was especially evident in the high hormonal follicular cycle phase.

### 4.1 Cognitive control across the menstrual cycle

In support of H1, a difference was found for the FL condition between the menstrual and follicular cycle phases, but no difference for the 10.13039/100010897NF or FR conditions (see Fig. 1). In keeping with H2, the multiple regression analysis with the hormone levels (estradiol and progesterone) showed a significant positive correlation between estradiol and FL scores. This is in line with two previous studies that found enhanced FL performance during the follicular cycle phase ([30]; Morris et al., 2015), and related increased estradiol levels (Hjelmervik et al. [30]. The FL condition is associated with high cognitive demands and the result could suggest recruitment of executive cognitive control abilities during the follicular cycle phase compared to the menstrual phase, and that estradiol facilitates such abilities. The results can also be seen in relation to other studies that investigated executive functions across the menstrual cycle or in relation to estradiol. Jacobs and D'Esposito [39] found increased performance on a verbal working memory task in the follicular-as compared to menstrual phase, especially in trials of high cognitive control demands. Similar findings have been reported in hormonal therapy studies, where administration of estradiol has positively impacted working memory [40] and cognitive inhibition [41,42].

### 4.2 Positive-schizotypy and language lateralization

As hypothesized (H3), a difference in language lateralization between high and low positive-schizotypy groups was found. While the low positive-schizotypy group showed pronounced left lateralized language processing, the high positive-schizotypy group showed more bilateral language organization. This pattern is in line with a previous study on male positive-schizotypy participants that used a similar paradigm with NF CV-syllables [23]. The effect was however driven by one of the cycle phases (discussed below). This might explain why atypical language lateralization in female positive-schizotypy participants is less pronounced [24]. Cycle phase could introduce more inter individual variability that has not been controlled for in previous studies ([24]; Castro and Pearson [22].

### 4.3 Positive-schizotypy, language lateralization and menstrual cycle phase

The hypothesis (H4) of more bilateral language lateralization (10.13039/100010897NF condition) in the high positive-schizotypy group during the high hormonal phase was supported by the Cycle phase x Positive-schizotypy group interaction (see Fig. 2). The interaction was driven by a significant difference between the high and low positive-schizotypy group in the follicular cycle phase that was not significantly present in the menstrual cycle phase. The low-positive-schizotypy group showed a pattern of increased REA (left hemispheric dominance) in the follicular cycle phase as compared to the menstrual cycle phase, which is in line with the findings of Cowell et al. [27]. The high-positive-schizotypy group showed a reversed pattern. The slightly left lateralized mean during the menstrual phase was shifted to a bilateral or slightly right lateralized mean in the high estradiol follicular cycle phase, in line with Hodgetts et al. [32]. The difference between high and low positive-schizotypy in cycle-related lateralization patterns can perhaps explain some of the inconsistencies in previous dichotic listening (NF) studies on the menstrual cycle. One may speculate that Cowell et al. [27] by chance have recruited participants with low degree of positive-schizotypy traits and therefore finds a cycle effect. While other studies not finding this effect ([30]; Morris et al., 2015), or finding the reversed pattern [32] could have included a larger portion of participants scoring higher on such traits.

It is important to highlight that while the FL condition showed menstrual cycle effects across all participants, the NF condition depended on positive-schizotypy traits. This provides further support that although FL is dependent on language lateralization, the cycle effect is probably related to the improved cognitive control abilities. Hence, there are likely two different cycle-related phenomena observed in the current study: one of cognitive control and one of language lateralization. One explanation for why the cycle effect on language lateralization (NF) is dependent on positive-schizotypy might lay in principles of interhemispheric inhibition – the inhibiting signals from dominant hemisphere onto the non-dominant hemisphere in order to reduce interference [43].

The NF results fit well with the hypothesis of altered interhemispheric inhibition across the menstrual cycle. The model of progesterone-mediated interhemispheric decoupling suggested reduced interhemispheric inhibition during the luteal cycle phase as compared to the menstrual phase [25]. The interhemispheric inhibition is reduced through suppression and enhancement of neuronal responses to respectively glutamate and GABA [44,45], leading to increased engagement of homotopic areas in contralateral hemisphere. There is however evidence also of an enhanced interhemispheric inhibition during the follicular cycle phase in comparison to luteal and menstrual cycle phase. For example, in a study of contralateral motor response it was found that Transcranial Magnetic Stimulation (TMS) of motor cortex showed cycle related differences in time length of motor response on the contra lateral side of the body, with the response being shorter in the follicular cycle phase as compared to the luteal and menstrual cycle phase [46]. This suggests stronger inhibition of the contralateral side during the follicular cycle phase. The increased right-ear advantage in the follicular cycle phase for low-positive-schizotypy group found in the present study could therefore suggest increased interhemispheric inhibition of the right hemisphere. This rationale could also explain the results for the high-positive-schizotypy group in the case of a right hemispheric dominance. An increased inhibition of the non-dominant left hemisphere would reduce the right ear responses and increase left ear responses.

#### 4.3.1 Neuroprotective effects of estradiol

While bilaterality is typical in schizophrenia and positive-schizotypy, it is unclear if it is a cause or a consequence of the disease. Hodgetts and Hausmann [33] argue that bilaterality could reflect a neurocompensatory mechanism similar to what have been suggested in other populations (e.g. elderly and dyslexia), and that the modulation of such asymmetries by estradiol could reflect neuroprotective effects. In short, neuroprotective effects of estradiol in schizophrenia are assumed due to less severe symptoms in women, especially during high estradiol cycle phases (for a review see Ref. [47]).

The results from the current study could be taken as support for such a notion [33], given that the bilaterality in high positive-schizotypy individuals is strongest during the high hormonal phase when protective effects are typically evident. As further support for this interpretation, the same cycle phase was associated with a reduction in hallucination proneness in a related sample [36], although in this study the role of positive-schizotypy was uncertain.

On a final note, although there might be underlying structural differences between high-low positive-schizotypy as previously suggested in schizophrenia (e.g. Ref. [20]), the results from the current study show that the functional outcome of these differences is highly dynamic/fluent rather than static, and probably dependent on the hormonal milieu at a given time.

### 4.4 Limitations

One limitation of the study is that sample size is relatively small. It should however be noted that the sample size of this difficult to recruit sample is in line with the majority of previous studies (e.g. Ref. [27]; Hjelmervik et al., 2015 [31]). Also, the current study replicated several previous findings (e.g., the effect of menstrual cycle on the FL-condition and the effect of more bilateral language in the high positive-schizotypy group), including those studies with larger sample sizes (Poreh, 1993). It is also important to note that strict methodology was applied. External hormonal validation ensured that women were tested in the correct cycle phase, and if not, excluded. This reduces measurement error and increases the statistical power in the study.

The between subject design has certain limitations. Individual changes in hemispheric asymmetries could have been more accurately assessed with a within-subject design that accounts for individual variability across the cycle [35], and a replication of the results in this respect is warranted. However, there are also advantages to adopt a between-subject design, such as (a) no order effects, (b) avoiding carry-over effects, (c) simpler design and analysis, (d) reducing the risk of participant drop out, (e) minimising the risk of participant bias. This might be the reason why between-subjects designs are also applied in menstrual cycle studies (e.g. Ref. [32,[48], [49], [50]]).

Lastly, the median split approach used to identify high and low positive schizotypy groups has limitations, which is an ongoing debate in the literature (e.g. Ref. [[51], [52], [53]]). The median split is frequently used in the research literature due to practical reasons such as simplifying the interpretation of variables, the analysis and the presentation of the results. However, it has been criticized for representing an arbitrary division that reduces individual variability [51]. The scores that lay close to the median is neither high nor low but is still categorized as either low or high. A stricter clinical division by preselecting individuals in the upper and lower range [54] could therefore be a useful approach in future studies. Furthermore, the most recent critique against the median split is that it might increase the chance of Type-1 error. However, Iacobucci et al. [52] has shown through statistical simulations that this is only a (minor) problem if the independent variables are related (but see Ref. [53]) – which was not the case in the current study.

## 5 Conclusion

The study aimed to test language lateralization in high and low positive-schizotypy individuals, and the modulatory effects of the menstrual cycle. The results confirmed an atypical language organization in female high positive-schizotypy individuals, with increased bilaterality in the high hormonal follicular phase. The opposite effect (stronger lateralization) was the case for low positive-schizotypy individuals during the follicular phase. The study suggests that the atypical language lateralization in high positive-schizotypy individuals is highly flexible and could potentially be related to neuroprotective effects of estradiol.

## CRediT authorship contribution statement

**Helene Hjelmervik:** Writing – original draft, Project administration, Methodology, Formal analysis, Conceptualization. **Josef J. Bless:** Writing – review & editing, Methodology, Data curation. **Julien Laloyaux:** Writing – review & editing, Methodology. **Kenneth Hugdahl:** Writing – review & editing, Funding acquisition. **Markus Hausmann:** Writing – review & editing, Methodology.

## Funding source

The contributions of coauthors H.H., JJB, and K.H. to the study was funded with a grant from 10.13039/100016882ERC #249516. The study was also funded by grants from Western Norway Regional Health Authority (Helse-Vest Samarbeidsorganet) grant #912045, both to K.H.

## Declaration of competing interest

The authors declare that they have no known competing financial interests or personal relationships that could have appeared to influence the work reported in this paper.
