# Supplementary figures and images for: Atypical language lateralization in positive schizotypy and modulating effects of the menstrual cycle
Source: Compr Psychoneuroendocrinol. 2025 Apr 2;22:100291. doi: 10.1016/j.cpnec.2025.100291 (PMC12018198; doi:10.1016/j.cpnec.2025.100291)

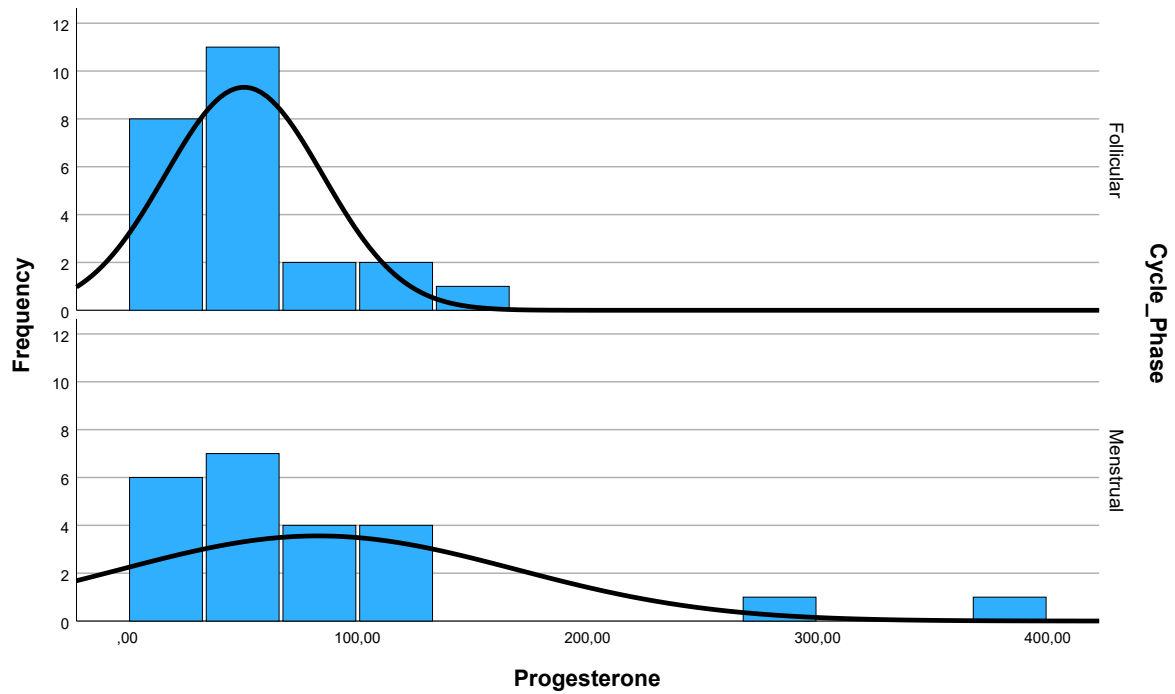

#### Hormone exclusion

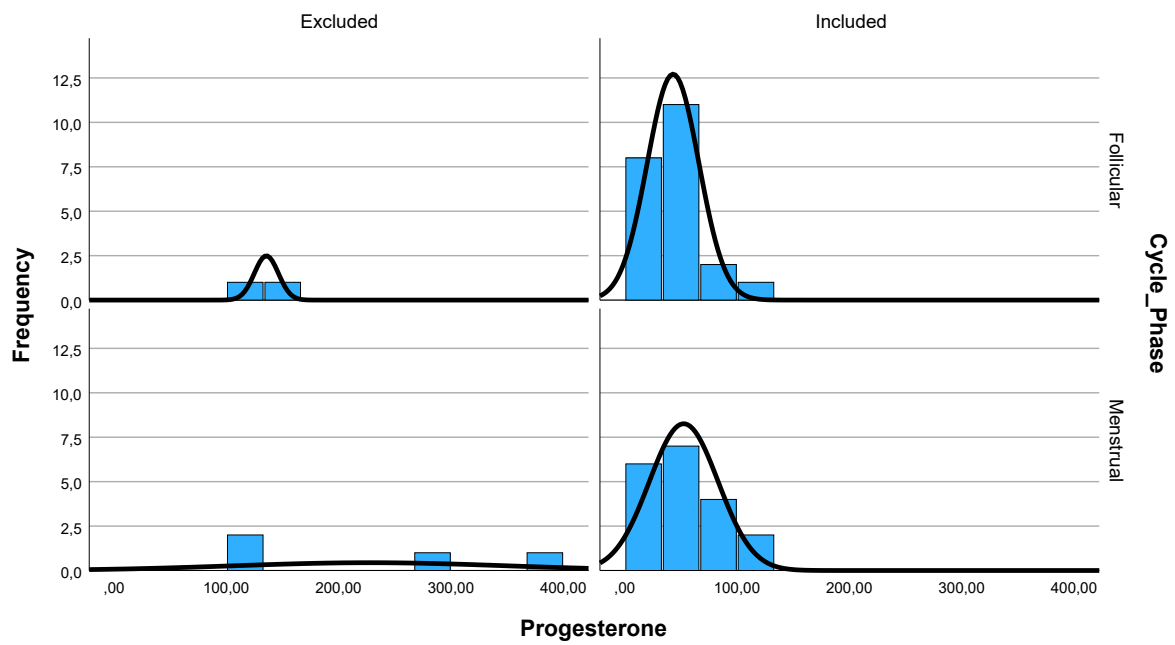

Supplement: Multimedia component 1 [file mmc1.pdf]

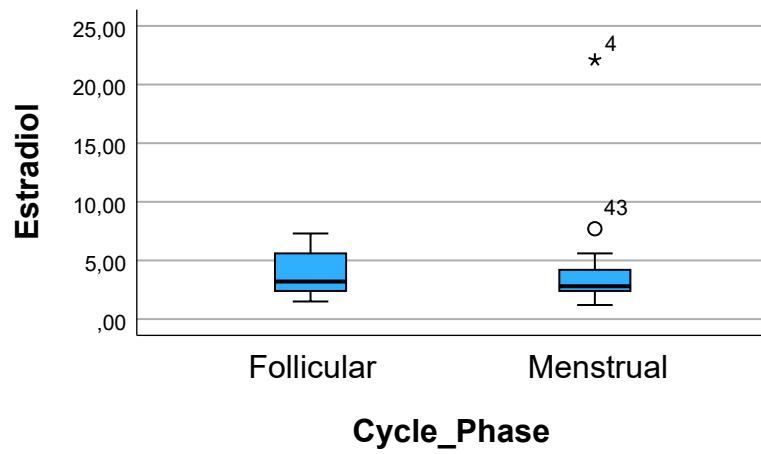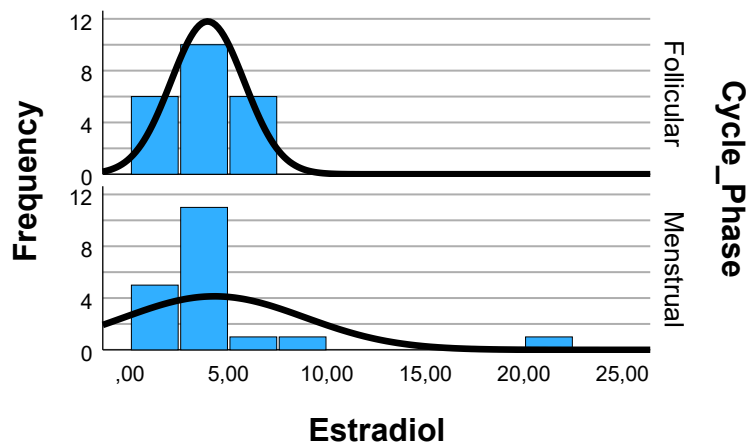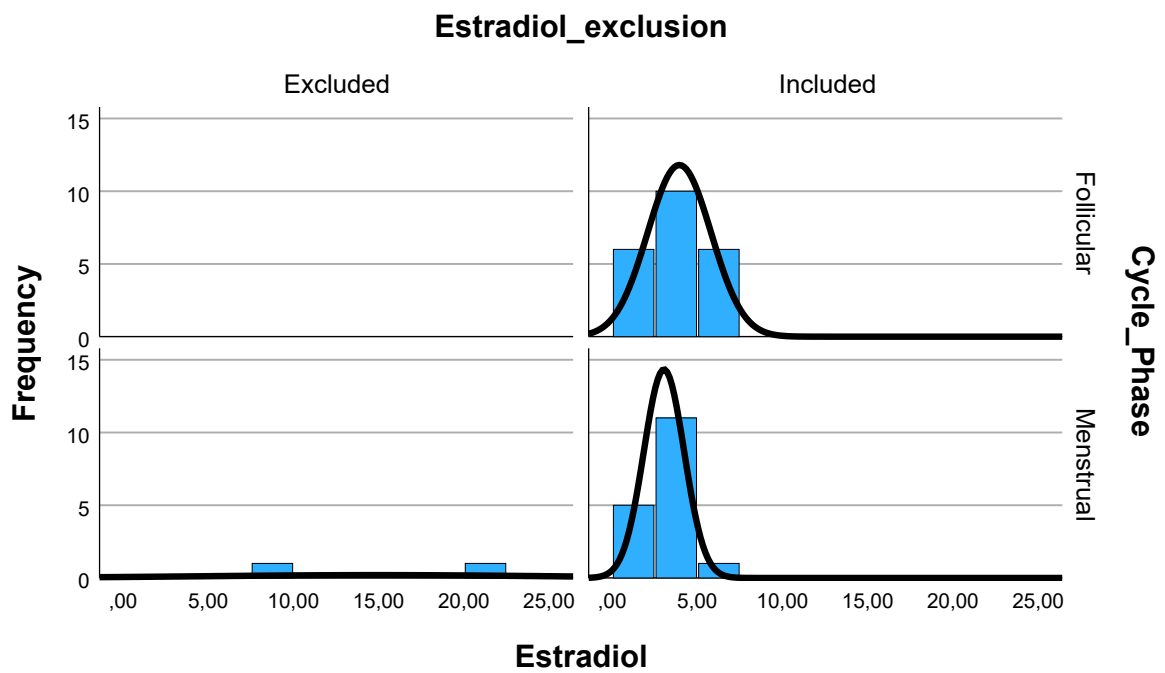

Supplement: Multimedia component 2 [file mmc2.pdf]

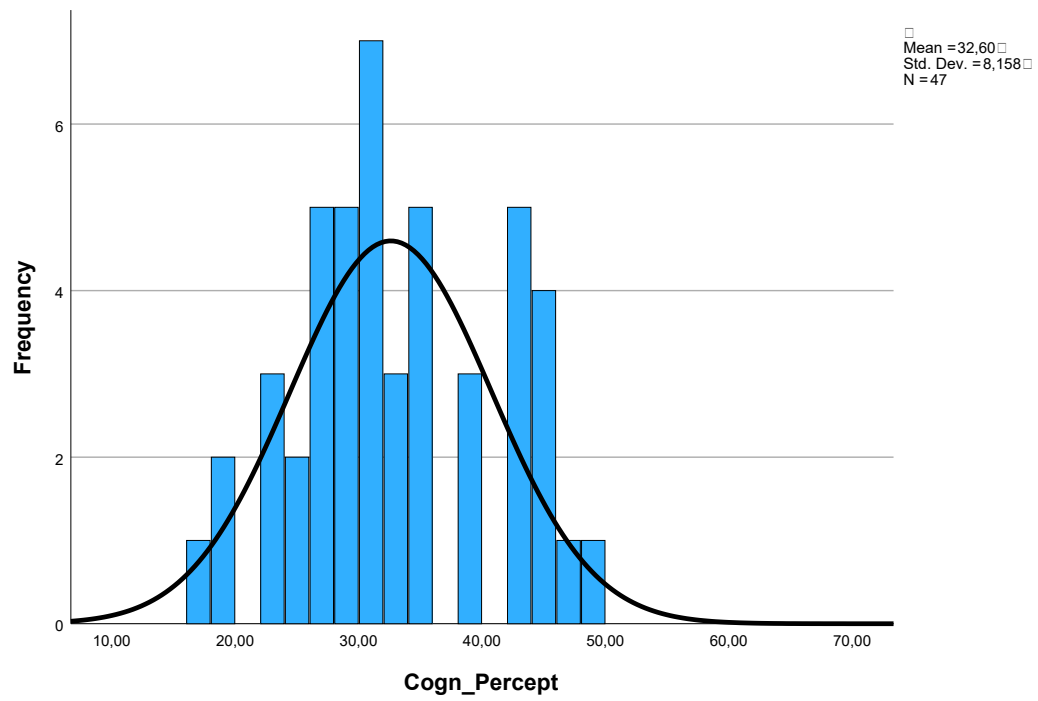

Supplement: Multimedia component 3 [file mmc3.pdf]
